# Protocol for setup and circadian analysis of inverted feeding in mice

**DOI:** 10.1016/j.xpro.2021.100701

**Published:** 2021-07-28

**Authors:** Haoran Xin, Rongfeng Huang, Meiyu Zhou, Xinyu Bao, Jianghui Chen, Fan Zeng, Xiaoqin Wan, Shifei Tong, Fang Deng, Min-Dian Li, Zhihui Zhang

**Affiliations:** 1 Department of Cardiology and the Center for Circadian Metabolism and Cardiovascular Disease, Southwest Hospital, Third Military Medical University (Army Medical University), Chongqing 400038, China; 2 Department of Cardiology, the Third Affiliated Hospital of Chongqing Medical University, Chongqing 401120, China; 3 Department of Pathophysiology, College of High Altitude Military Medicine, Key Laboratory of Extreme Environmental Medicine, Ministry of Education of China, and Key Laboratory of High Altitude Medicine, PLA, Army Medical University (Third Military Medical University), Chongqing 400038, China

**Keywords:** Bioinformatics, Metabolism, Model Organisms, Molecular Biology, Gene Expression, Behavior, Systems biology

## Abstract

Inverted feeding is a paradigm to study synchronization of circadian clocks by feeding rhythm in tissues more directly. Here, we provide a protocol for performing inverted feeding in mice and analyzing circadian rhythmicity in mouse tissues. We describe setting up inverted feeding and performing tissue dissection, followed by RNA extraction and gene expression analysis, and lastly R software-based analysis of circadian rhythmicity. This protocol can be combined with the use of CircaMetDB database for mechanistic studies of inverted feeding.

For complete details on the use and execution of this protocol, please refer to Xin et al. (2021).

## Highlights

• Set up inverted feeding (IF) to evaluate clock synchronization by meal time
• Hands-on tutorial of circadian rhythmicity detection and comparison in R
• Protocol combined with CircaMetDB resource can be used for mechanistic study of IF

## Graphical abstract

**Figure:**
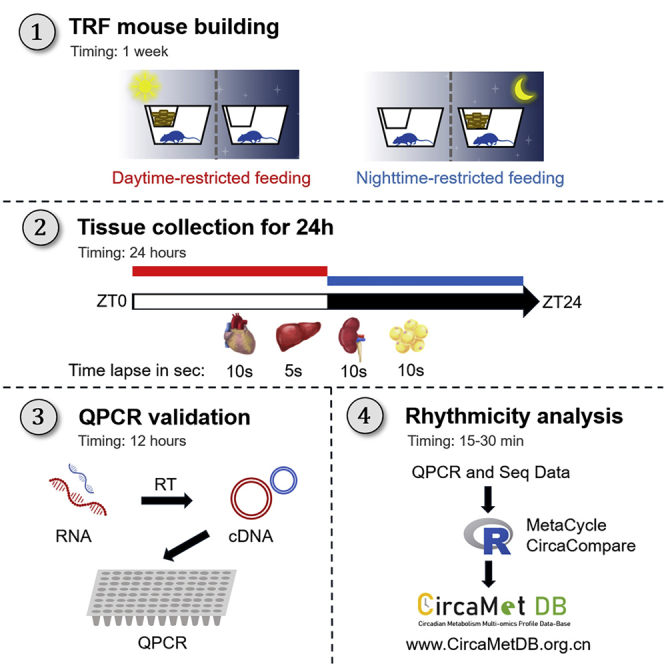

## Before you begin

The protocol below describes the specific steps for a circadian study covering 24 h in regular light-dark cycles. However, we have also used this protocol in continuous light condition with minor modifications.

### Light-tight cabinet setup

**Timing: 1 h**

**Figure 1:**
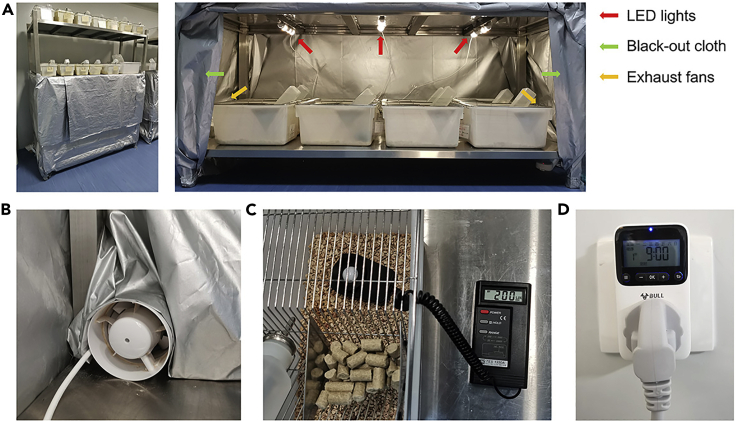
Light-tight cabinet set-up (A) A light-tight cabinet is installed with LED lights (red arrow), black-out cloth (green arrow), and exhaust fans (yellow arrow). (B) Exhaust fan installed in the rear-end bottom of the racks. (C) Light meter showing the reading of light intensity. (D) Programmable timer switch showing the lighting schedule.

1. Prepare materials required for setting up light-tight cabinets (Figure 1A), such as black-out cloth, LED lights, a light meter, programmable timer switches, and exhaust fans.
  a. Install LED lights on the top of the breeding racks, and connect to the electric power.
  b. Install exhaust fans on the bottom of the breeding racks, and connect to the electric power (Figure 1B).
  c. Encircle the racks with black-out cloth to control illumination.
  d. Adjust the LED luminescence to be within the range of 190–210 lux (average light intensity 200 lux) based on the reading from the light meter placed on the bedding of the cage (Figure 1C).
  e. Set up the light-on/off schedule on the programmable timer switch. Light-on time is 9:00 (Zeitgeber time 0, ZT0). Light-off time is 21:00 (ZT12) (Figure 1D).

**CRITICAL:** When handling mice or performing dietary intervention, open the cloth in the front.

### Animal grouping and randomization

**Timing: 1–2 weeks**

2. Acquire 60 specific pathogen-free C57BL/6J female mice from a certified vendor, and assign four biological replicates per time point per treatment. The following procedure also applies to males.
3. Acclimate C57BL/6J female mice at the age of 7 weeks to a 12 h light:12 h dark cycle with free access to normal chow food and water (ad libitum) for at least one week.
4. Assign animals randomly in a 1:1 ratio to time-restricted feeding groups. Then, balance the body weight statistics between groups before subjecting to time-restricted feeding.
  a. Identify a mouse by an ear tag and weigh the body weight.
  b. Create a new Excel table, enter the mouse identity number and body weight, and sort ascendingly.
  c. Generate a random number for each mouse.
  d. Assign mice with odd random numbers to nighttime-restricted feeding (NRF) group, and those with even random numbers to daytime-restricted feeding (DRF) group.
  e. Put four mice per cage based on grouping.
5. Determine the statistics of body weights, and adjust the grouping so that there is no statistical difference between groups (t-test, p-value > 0.08).
  a. Perform t-test of body weight between DRF and NRF.
  b. If p-value > 0.08, proceed to next steps.
  c. If p-value < 0.05, swap some mice between DRF and NRF to balance the mean body weight, so that the new p-value > 0.08.
  d. If 0.05 < p-value < 0.08, carefully check the distribution of body weight and SEM, swap mice between groups to adjust the p-value to be > 0.08.
  e. Finalize the mouse grouping based on the adjusted assignment.

**CRITICAL:** 56 mice are needed for one experiment (DRF vs NRF). Acquirement of 60 ensures 2 backup mice per group.

***Note:*** One advantage of using females in these studies is that randomization is easy to operate since females are much less aggressive in territory behaviors than males.

***Note:*** The difference of the mean body weight (around 18.5 grams for 7-week-old female C57BL/6J mice) of mice between DRF and NRF groups is within 0.3 gram (1.5%), the standard error of the mean (SEM) for each group is within 0.6 gram.

***Note:*** In our original study, we compared the effects of inverted feeding in both males and females. Please refer to Xin et al., 2021 for details. Briefly, the phenotype is robust in liver and adipose tissue between male and female. Inverted feeding disrupted the rhythms of the majority of selected core clock genes in heart and kidney in males, whereas the rhythms were preserved in females (Xin et al., 2021). Very recently, Manella et al. have used Alb-Cre+ male mice as the wildtype control, and recapitulated our observations in males under a 30-day inverted feeding, or daytime feeding as they called it (Manella et al., 2021). Sexual dimorphism is emerging as a key factor in studying circadian rhythm (Anderson and FitzGerald, 2020; Weger et al., 2019).

### Sample collection

**Timing: 1 h**

6. Prepare the required tools, materials and reagents before tissue dissection (see Table 1).

**Table 1:** Tools, materials, and reagents for tissue dissection

Equipment or reagent	Function
Tissue scissors	Dissect tissues
Micro tweezer	Dissect tissues
Filter paper	Drain surface liquid from tissues
Nuclease-free EP tube, 1.5 mL	Store tissues
Liquid nitrogen	Snap freeze tissues
75% ethanol	Prevent hair fall and maintain a clean environment
PBS buffer	Rinse tissues

**CRITICAL:** Surgical instruments need to be sterilized and disinfected before use.

## Key resources table

**Table:** 

REAGENT or RESOURCE	SOURCE	IDENTIFIER
**Critical commercial assays**		

Eastep® Super Total RNA Extraction Kit	Promega	Cat# LS1040
GoScript™ Reverse Transcription Mix	Promega	Cat# A2801
iTaq™ Universal SYBR ® Green Supermix	Bio-Rad	Cat# 1725124
Microseal ‘B’ seal Seals	Bio-Rad	Cat# MSB1001

**Deposited data**		

Analyzed data (RT-qPCR, RNA-seq, code)	(Xin et al., 2021)	Mendeley Data https://doi.org/10.17632/mb25x9t4m7.1
Transcriptomics, visceral adipose tissue	(Xin et al., 2021)	CNGBdb:CNP0001638

**Experimental models: Organisms/strains**		

Mus musculus, C57BL/6J, male, 7 weeks, specific pathogen free	Hunan SJA Laboratory Animal Co. Ltd	http://www.hnsja.com/product/7.html
Mus musculus, C57BL/6J, female, 7 weeks, specific pathogen free	Hunan SJA Laboratory Animal Co. Ltd	http://www.hnsja.com/product/7.html

**Oligonucleotides**		

Primers: Arntl Forward: CTTGCAAGCACCTTCCTTCC Reverse: GGGTCATCTTTGTCTGTGTC	(Xin et al., 2021)	N/A
Primers: Per2 Forward: ATGCTCGCCATCCACAAGA Reverse: GCGGAATCGAATGGGAGAAT	(Xin et al., 2021)	N/A
Primers: Nr1d1 Forward: TACATTGGCTCTAGTGGCTCC Reverse: CAGTAGGTGATGGTGGGAAGTA	(Xin et al., 2021)	N/A
Primers: Dbp Forward: CGTGGAGGTGCTAATGACCTTT Reverse: CATGGCCTGGAATGCTTGA	(Xin et al., 2021)	N/A
Primers: 1110038B12Rik-201 Forward: GCACAATGGGATTTGAGGACAC Reverse: GACAAAGGGCTGGCTCTCAT	(Xin et al., 2021)	N/A
Primers: 2410006H16Rik-201 Forward: TGCTCTTCTCGCTCGTTGAG Reverse: TTGTCAACGTCCCGTCAGG	(Xin et al., 2021)	N/A
Primers: AC157910.3-202 Forward: GTGACTCTGTTCCCTGGTGA Reverse: TGTGGAGTCACTTTGCTGCA	(Xin et al., 2021)	N/A
Primers: Gm45866-201 Forward: AAACAGCCACCACACGGTAC Reverse: CCCTTCCAGTTGGCCTTTGA	(Xin et al., 2021)	N/A

**Software and algorithms**		

R Project for Statistical Computing	https://www.r-project.org	v4.0.2 RRID:SCR_001905
RStudio	http://www.rstudio.com	v1.2.5033
R package: CircaCompare	(Parsons et al., 2020)	v0.1.0
R package: MetaCycle	(Wu et al., 2016)	v1.2.0
Graphpad Prism 8.0.1	https://www.graphpad.com	v8.0.1
Mendeley Desktop	Elsevier	v1.19.4
Excel 2019	Microsoft	16.0.14026.20202

**Other**		

Normal chow diet (rodent maintenance diet)	Hunan SJA Laboratory Animal Co. Ltd	http://www.hnsja.com/product/13.html
Exhaust fan	TonDa&Keiji Co. LTD	Cat# TDF-150A
Programmable light switch	Bull Group Co., Ltd	Cat# GND-1
Black-out cloth	Taobao.com/Shaoxing Jiuzun Co. Ltd	N/A
LED lights	OPPLE Lighting Co. Ltd	Cat# KUBI-3.5W
Light meter	TES Electrical Electronic Corp	Cat# 1330A
Multiplate PCR plate 96-well, white	Bio-Rad	Cat# MLL9651
Tissue scissors	Beyotime	Cat# FS001
Micro tweezer	Beyotime	Cat# FS027
Filter paper	Beyotime	Cat# FFT08
Nuclease-free EP tube, 1.5 mL	NEST	Cat# 615601
Liquid nitrogen	Southwest Hospital	N/A
75% Ethanol (diluted from AR ethanol absolute)	Chuandong Chemical Group	AR
PBS buffer	Beyotime	Cat# C0221A
Cryogenic grinding system	Shanghai Jingxin Industrial Development Co. Ltd	JXFSTPRP-CL
NanoDrop 2000C Spectrophotometer	Thermo Scientific	NanoDrop 2000C
Real-time PCR detection system	Bio-Rad	CFX96
PCR thermal cycler	Bio-Rad	C1000

## Materials and equipment

Here we provide additional information regarding equipment setup for reverse transcription (RT), real-time quantitative PCR (RT-qPCR), and the recipe for preparing master premix of RT-qPCR.

**Table:** Equipment setup for reverse transcription

Steps	Temperature	Time
1	25°C	5 min
2	42°C	60 min
3	70°C	15 min
4	4°C	∞

**Table:** Equipment setup for real-time quantitative PCR

Steps	Temperature	Time	Cycles
1	95°C	3 min	1
2	95°C	10 s	40
3	60°C	30 s	
4 Melting curve	65->95°C , by 0.5°C	5 s every 0.5°C increment	1

**Table:** Recipe for qPCR premix

Reagents	Final concentration	Amount
Nuclease-free water	n/a	160 μL
Forward primer (10 μM)	0.625 μM	40 μL
Reverse primer (10 μM)	0.625 μM	40 μL
SYBR master mix (−20°C for 12 months)	n/a	400 μL
**Total**	**n/a**	640 μL

**CRITICAL:** Keep the SYBR master mix in a −20°C constant temperature freezer for 12 months and avoid light. For short-term storage, it can be stored at 4°C for up to 8 h.

***Alternatives:*** SYBR master mix is available from many vendors, including Bio-Rad and Thermo, but it has to be compatible with the real-time PCR detection system.

***Note:*** This recipe is used for a typical test size of 80. Technical replicate is one for samples, and two for standards and blank control. Ideally, technical replicate should be two for samples when 384-well block is available for the qPCR instrument.

## Step-by-step method details

To set up inverted feeding paradigm, we used daytime-restricted feeding (DRF) to introduce inverted feeding to mice and nighttime-restricted feeding (NRF) as a control. This procedure is written for a 36-day time-restricted feeding regimen, but it is commonly applied for as short as 7 days in Xin et al., 2021.

### Time-restricted feeding regimens

**Timing: 36 days**

**Figure 2:**
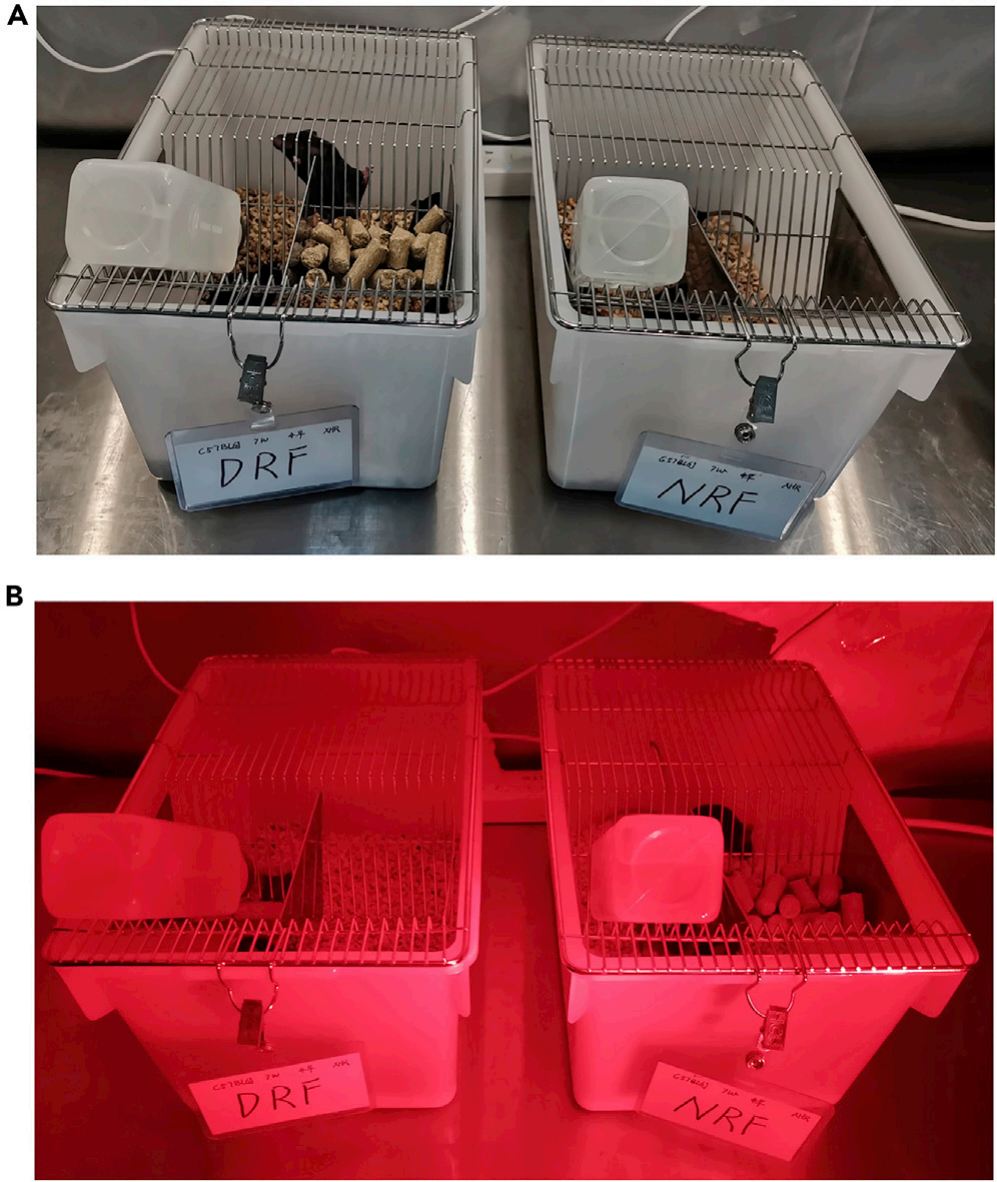
Time-restricted feeding regimens (A) Appearance of DRF and NRF cages in the light phase. Food pellets are placed on the hopper of a DRF cage but not on that of a NRF cage. (B) Appearance of DRF and NRF cages in the dark phase. Food pellets are placed on the hopper of a NRF cage but not on that of a DRF cage. Red lamp is on when operating in the dark phase.

1. At the end of acclimation and group assignment, start the time-restricted feeding regimens in the light-tight cabinets.
  a. Place big food pellets (5 pellets per mice, 20–30 pellets per cage) on the food hopper.
  b. DRF group has access to food for 12 h from ZT0 (9:00) to ZT12 (21:00), whereas NRF group has access to food for 12 h from ZT12 (21:00) to ZT24 (ZT0, 9:00). Mice have access to water ad libitum (Figure 2).

***Optional:*** To determine whether removal of time cues from the suprachiasmatic master clock affects food entrainment in peripheral tissues, constant light condition is applied simultaneously with the 7-day daytime-restricted feeding. To set up constant light, the LED lights are adjusted to allow 500 lux at the bedding of mouse cages (Chen *et al.*, 2008), and the light-off time on the timer switch is set to the same as the light-on time (i.e., 9:00).

***Optional:*** To determine the long-term effects of inverted feeding on tissue clocks and biology, the regimen can be extended. The longest regimen in our study lasts for 36 days.

**CRITICAL:** Four mice are assigned to one cage. Clearly label the NRF/DRF assignment on the cage registration sheet (Figure 2). Check the timer switch for time accuracy at the start of the experiment and at least once during the experiment.

**CRITICAL:** When performing time-restricted feeding under 12 h light:12 h dark cycles, the 7-day regimen is normally applied and sufficient to entrain the liver clock (Damiola et al., 2000; Stokkan et al., 2001; Hara et al., 2001). We tested the time of time-restricted feeding for up to 36 days and found consistent phase entrainment in the four tested peripheral tissues, i.e., heart, kidney, liver, and adipose tissue (Xin et al., 2021).

2. Record the food weight daily and body weight weekly.
  a. Weigh the food weight at ZT0 and ZT12 and work in the dark phase under a red lamp.
  b. Calculate food consumption per cage. Since four mice are assigned per cage, average food consumption by a factor of four for food intake per day per mouse.
  c. Weigh the body weight at ZT0.

**CRITICAL:** To ensure normal light-dark cycles, it is crucial to work in the dark phase under a red lamp when weighing and changing food pellets.

**CRITICAL:** The success of time-restricted feeding regimens depends on the scheduled restriction of food access. Since mice occasionally smash food pellets, the smashed food power fallen to the bedding would violate food restriction, if it were not removed timely. Measurement of food consumption would also indicate the metabolic status of mice. Thus, during dietary regimens, look for smashed food in the cage daily to avoid violation of time restriction and mis-calculation of food consumption. Big food pellets are fed to mice to prevent from food smashing. In addition, the action of food restriction is normally completed within 10 minutes of light switch. Don't forget to keep the water adequate.

***Optional:*** When performing time-restricted feeding regimens within 7 days, it is optional to monitor body weight. It is a routine in our laboratory to track body weight every week, and body weight is weighed at ZT0 in the beginning and end of a 7-day regimen.

### Tissue dissection

**Timing: 24 h**

This section details the optimized steps to dissect and handle mouse tissues.

3. Euthanize the mice by cervical dislocation every 4 h over a complete 24 h cycle.
  a. The number of biological replicates per treatment per time-point is four.
  b. Time points are Zeitgeber Time (ZT) 0, 4, 8, 12, 16, 20, 24 h. ZT24 is the start of the next day.
  c. Euthanize a mouse by cervical dislocation, fix it on the dissection plan with pins, identify the thorax and abdomen.
  d. Spray the surface of mice with 75% ethanol.
  e. Cut a horizontal incision at the abdomen by scissors and tear the skin towards head and tail, respectively, to expose the mouse's thorax and abdomen.

**CRITICAL:** During the dark phase, red lamp is used for illumination to avoid disruption of light-dark cycle (Figure 2). The mouse is euthanized under the red lamp in the dark before relocating to the operating hood for tissue dissection.

**Figure 3:**
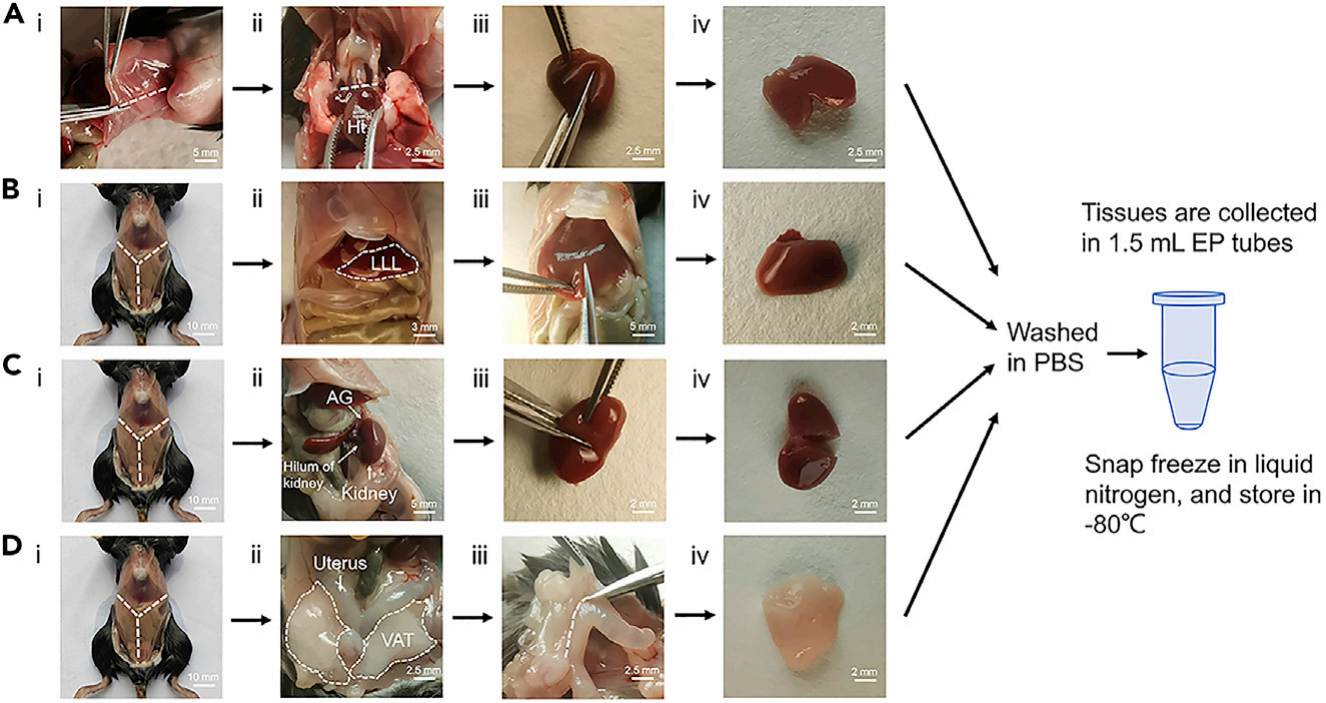
Tissue dissection (A) Dissection procedure for heart. (B) Dissection procedure for liver. (C) Dissection procedure for kidney. (D) Dissection procedure for VAT. Ht, heart; LLL, left lobe of liver; AG, adrenal gland; VAT, visceral adipose tissue or perigonadal adipose tissue. Scale bars are shown in white and range from 2 to 10 mm as indicated.
  b. Dissect liver tissue (Figure 3B).
    i. Make a Y-shaped incision on the peritoneum to expose all abdominal organs.
    ii. Identify the left lobe of liver (LLL) in the upper left side of the mouse's abdominal cavity.
    iii. Cut about 40 mg of the LLL, wash with PBS briefly, and wipe the liver with filter paper. Be careful not to cut the gallbladder to avoid contaminating the tissue with bile.
    iv. Quickly put the tissue into the labeled tube and freeze immediately in liquid nitrogen, store at −80°C.
  c. Dissect kidney tissue (Figure 3C).
    i. Make a Y-shaped incision on the peritoneum to expose all abdominal organs.
    ii. Set off the intestine and find the kidney near the back of the mouse, cut out the kidney at the hilum of the kidney, strip the adrenal gland (AG) and perirenal fat.
    iii. Cut the kidney along the lateral edge, wash with PBS briefly, and wipe the kidney with filter paper.
    iv. Quickly put the tissue into the labeled tube and freeze immediately in liquid nitrogen, store at −80°C.
  d. Dissect visceral adipose tissue (Figure 3D).
    i. Make a Y-shaped incision on the peritoneum to expose all abdominal organs.
    ii. Find the Y-shaped uterus in the lower abdomen near the anus. The white soft tissue around the uterus is visceral adipose tissue (VAT, also known as perigonadal adipose tissue).
    iii. Cut the VAT along the edge of the uterus, wash with PBS briefly, and wipe with filter paper.
    iv. Quickly put the tissue into the labeled tube and freeze immediately in liquid nitrogen, store at −80°C.

4. Dissect mouse tissues in the following order, which gives high priority to tissues with high metabolic demand like heart.
  a. Dissect heart tissue (Figure 3A).
    i. Cut off the diaphragm and ribs along the sides of the mouse body, set off the front wall of the thorax, and make the heart visible.
    ii. Cut out the entire heart at the junction of the aorta and the heart.
    iii. Cut the heart chambers, wash with PBS briefly, and wipe the heart with filter paper to deplete residual blood.
    iv. Quickly put the tissue into the labeled tube and freeze immediately in liquid nitrogen, store at −80°C.

**CRITICAL:** The collection time for these tissues should be within 5 minutes per mouse, and the blood should be washed away as much as possible. Moreover, the label on the EP tubes for tissue storage should be checked carefully to avoid misplacement.

### RNA extraction and cDNA preparation

**Timing: 8 h**

The steps below apply to one mouse tissue. Briefly, tissue RNA is isolated using Eastep Super Total RNA Extraction Kit (Promega). Complementary DNA (cDNA) is synthesized using the GoScript Reverse Transcription Mix (Promega). cDNA is amplified and analyzed using iTaq universal SYBR Green Supermix (Bio-Rad) and the Bio-Rad CFX96 Real-Time PCR Detection System (Bio-Rad).

5. RNA extraction from mouse tissues.
  a. Mouse tissues are homogenized in a Cryogenic Grinding System (Shanghai Jingxin JXFSTPRP-CL).
    i. Grinding condition is set to a power of 65 hertz for 2 min at 4°C.
  b. 30–40 mg of samples from kidney, heart, and a trunk from the left lobe of liver sampled from the same region are used for RNA extraction. 80–100 mg of adipose tissue is used for RNA extraction.
  c. Tissue homogenates are subjected to RNA extraction using Eastep Super Total RNA Extraction kit.
    i. Detailed steps can be found in the link: RNA extraction using Eastep® Super Total RNA Extraction kit.
6. Perform reverse transcription to prepare complementary DNA (cDNA) samples.
  a. Measure the concentration of extracted RNA in a spectrophotometer.
  b. Aliquot 2,000 ng of total RNA for reverse transcription reaction.
  c. Detailed steps can be found in the link: cDNA is synthesized using the GoScript™ Reverse Transcription Mix.
  d. Equipment setup for RT is described in the materials and equipment section.

**CRITICAL:** Nuclease-free tubes, tips, dissection tools are important for all these steps of tissue collection, RNA isolation and cDNA synthesis.

**CRITICAL:** All steps are performed on ice or in a prechilled 4°C environment.

### Real-time quantitative PCR

**Timing: 1–2 days**

We applied the standard curve method to quantify mRNA levels in this study. The 2-ΔΔCt method is also applicable.

**Figure 4:**
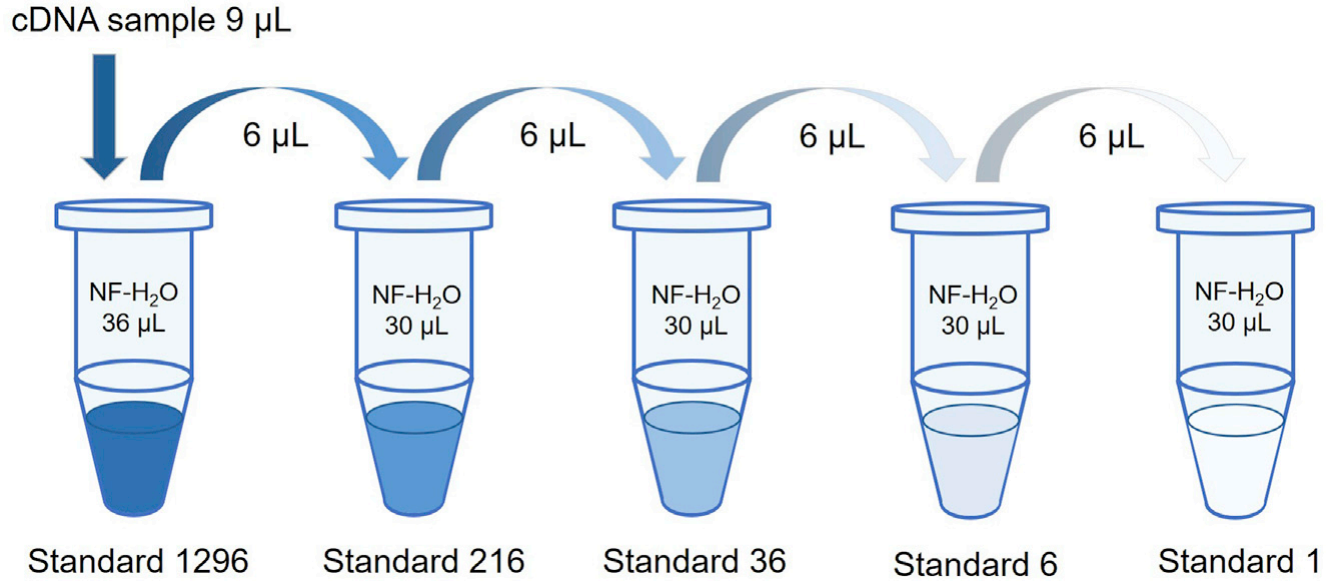
Preparation of Standards for RT-qPCR 9 μL cDNA solution in total is mixed with 36 μL nuclease-free water (designated as Standard 1296). 6 μL is aliquoted from Standard 1296 and mixed with 30 μL nuclease-free water (designated as Standard 216). This is done serially to prepare Standard 36, 6, 1. Nuclease-free water is designated as Standard 0 and serves as blank control. NF-H_2_O, nuclease-free water.

7. Prepare the diluted cDNA samples and standards.
  a. Dilute cDNA samples 20 times, i.e., 2 μL reverse transcription (RT) reaction + 38 μL nuclease-free water.
  b. Next, prepare the Standard samples for plotting the standard curve to estimate replication efficiency and relative quantification.
  c. To prepare the first Standard (1,296 arbitrary units), sample and combine 3 μL inputs from RT reactions at three time points, i.e., ZT0, ZT8, ZT16 from NRF group, when circadian clock genes Arntl, Nr1d1, Per2, reach peak expression. (See CRITICAL below)
  d. Mix these 9 μL RT reactions with 36 μL nuclease-free water.
  e. Dilute Standard 1296 at a 1:6 ratio serially to Standard 1 (Figure 4).

**Pause point:** cDNA samples and standards can be kept at −20°C for months, but avoid frequent freeze and thaw.

**CRITICAL:** Ideally, one sample from each time point should be added to the pool to make the first and most concentrated Standard sample. In our experience, pooling samples from three time points in the NRF group, i.e., ZT0, ZT8, and ZT16, is sufficient to cover the quantity of major clock genes.

8. Prepare the premix for 80 reactions.
  a. Refer to Recipe for qPCR Premix in the materials and equipment section for details.
  b. Detailed steps can be found in the link: RT-qPCR using iTaq Universal SYBR® Green Supermix kit.
9. Add 2 μL cDNA sample to a 96-well PCR plate, and then add 8 μL premix to form a 10 μL reaction system.
10. Seal the PCR plate. Briefly spin down in a benchtop centrifuge.

**Pause point:** The PCR plate can be wrapped in foils and store in 4°C for up to 5 h.

11. Run the RT-qPCR reaction in a real-time PCR detection system.
  a. Equipment setup for RT-qPCR is described in the materials and equipment section.

***Note:*** Mix well by gently pipetting up and down 10 times. Ensure that the amplification efficiency is 90%–110%, and R2 ≥ 0.98. Otherwise, use the 2-ΔΔCt method for quantification.

## Expected outcomes

### Expected outcomes of RNA extraction

The quality control is usually in the range of 2.0–2.1 (A260/A280) and 1.85–2.3 (A260/A230). Concentration of RNA extract is variable among tissue types. Refer to Table 2 for tissue-specific outcomes.

**Table 2:** Quality control and concentration of tissue RNA extract

Tissue	A260/280	A260/230	Concentration (ng/μL)
Liver	2.08–2.15	2.00–2.30	500–900
Heart	2.02–2.08	1.90–2.22	250–400
VAT	2.03–2.07	1.85–2.17	300–500
Kidney	2.06–2.12	1.95–2.27	800–1200

### Expected outcomes from RT-qPCR

**Figure 5:**
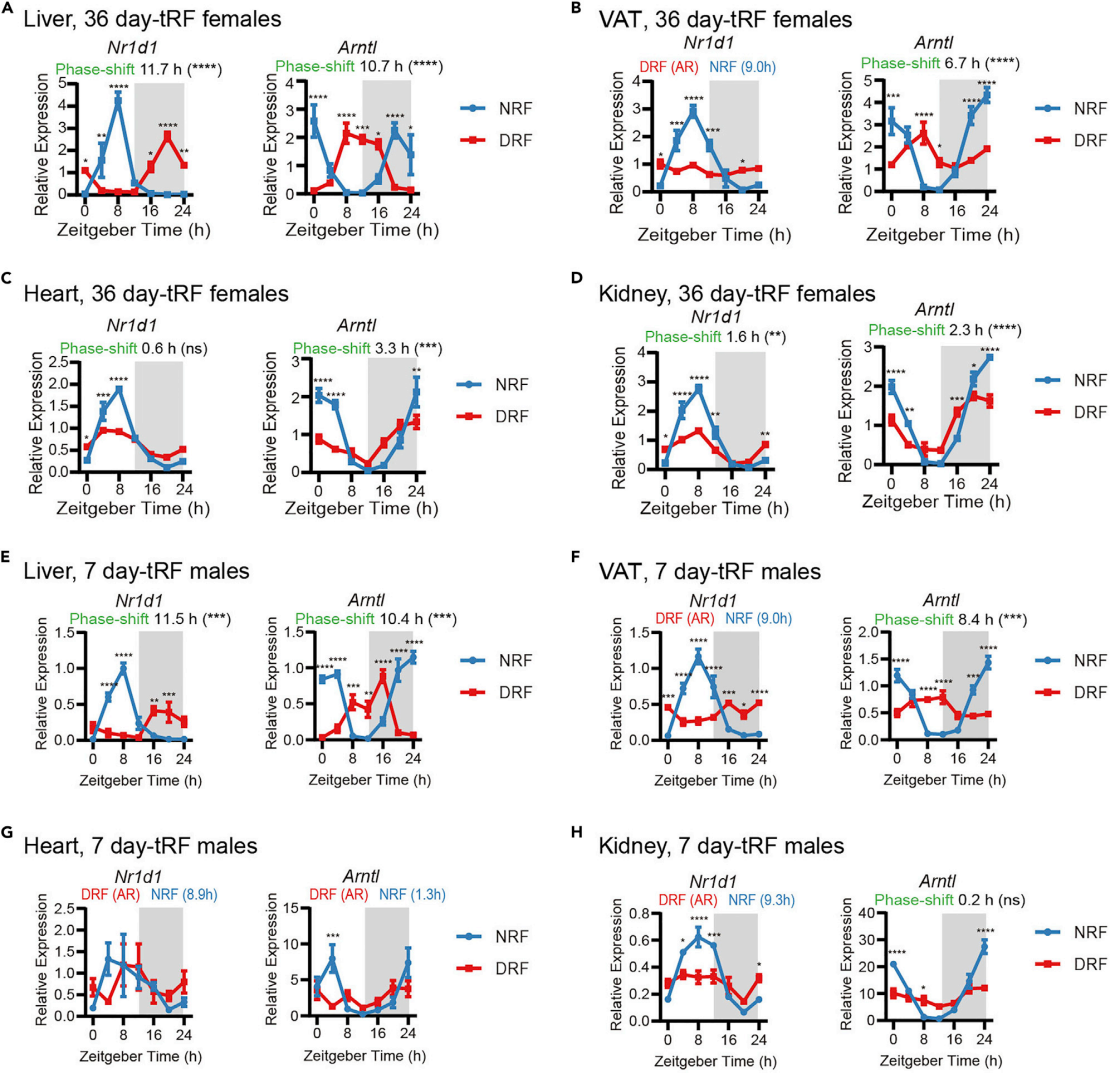
Diurnal expression of circadian clock genes in mouse tissues subjected to time-restricted feeding (tRF) (A–D) Diurnal expression of clock genes Nr1d1 and Arntl in female mouse liver (A), VAT (B), heart (C), and kidney (D) subjected to 36-day tRF. (E–H) Diurnal expression of clock genes Nr1d1 and Arntl in male mouse liver (E), VAT (F), heart (G), and kidney (H) subjected to 7-day tRF. Data were represented as mean ± sem (n = 4). Multiple t tests with Bonferroni correction; ns, p > 0.05, ∗p < 0.05, ∗∗p < 0.01, ∗∗∗p < 0.001, ∗∗∗∗p < 0.0001. Phase was calculated by MetaCycle (meta2d_phase). Phase shifts were represented by the absolute value of phase difference between DRF and NRF in a 12-h window, and calculated by CircaCompare, which provides a p-value for phase-shift. Figure reprinted with permission from Xin et al., 2021.

**Figure 6:**
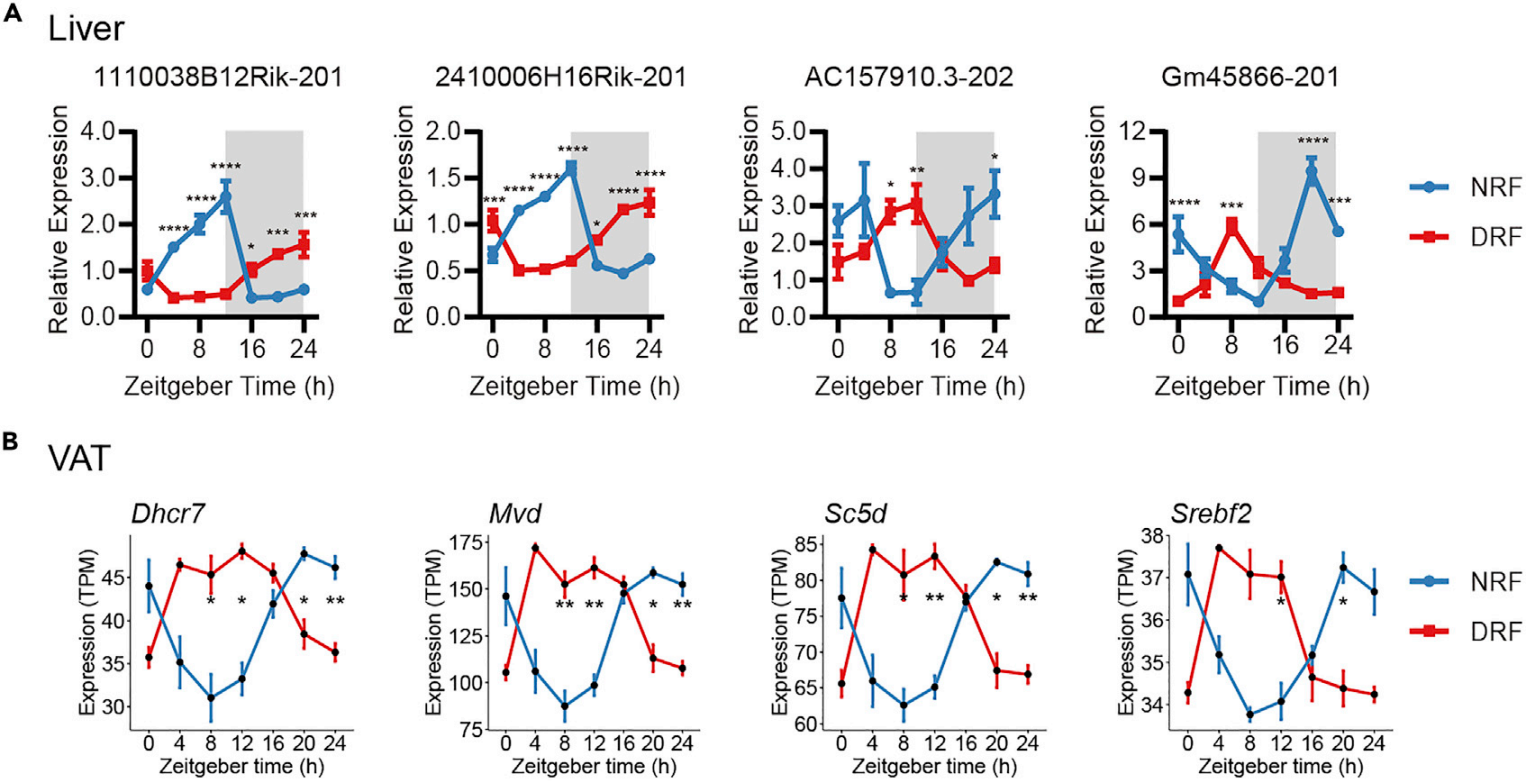
Diurnal expression of long non-coding RNAs and metablic genes in mouse tissues subjected to 7-day tRF (A) Diurnal expression of long non-coding RNAs in liver subjected to 7-day tRF. (B) Diurnal expression of metabolic genes involved in cholesterol biosynthesis in VAT subjected to 7-day tRF. Data were represented as mean ± sem (n = 4). Multiple t tests with Bonferroni correction; ns, p > 0.05, ∗p < 0.05, ∗∗p < 0.01, ∗∗∗p < 0.001, ∗∗∗∗p < 0.0001. Figure reprinted with permission from Xin et al., 2021.

Diurnal expression of clock genes in different peripheral tissues or sexes (Figure 5), circadian long non-coding RNAs (Figure 6A), and clock output genes such as cholesterol biosynthesis (Figure 6B), could be profiled from these samples. The CircaMetDB database (www.circametdb.org.cn) could serve as a reference to inquire genes of interest.

## Quantification and statistical analysis

### Determination of circadian rhythmicity

*This quantification and statistical analysis is applied to female/male mouse peripheral tissues subjected to tRF with different treatment time. It is also applied to other tRF conditions in the original study* (Xin et al., 2021).

**Figure 7:**
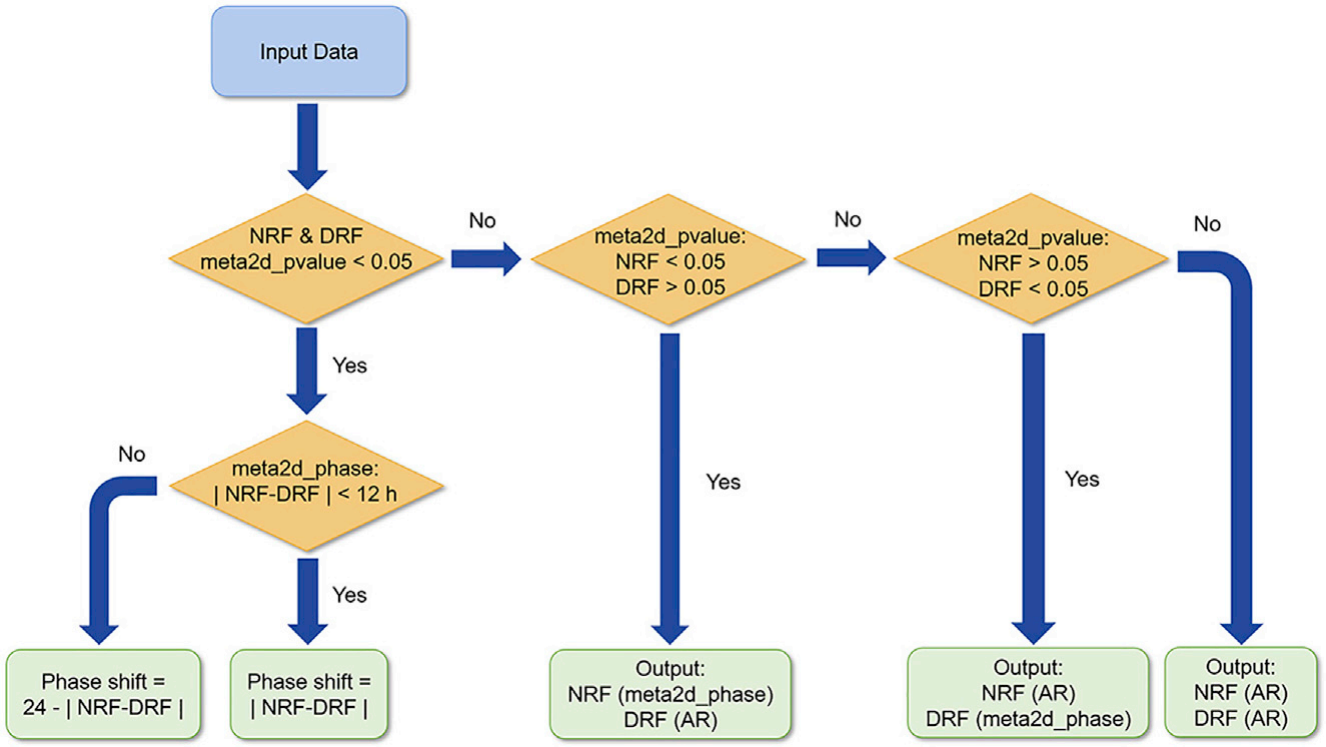
Algorithm for determining circadian rhythmicity and phase shift In the results of Meta2d analysis, if meta2d_pvalue < 0.05 (or meta2d_BH.Q < 0.05 if analyzing RNA-seq) for both tRF regimens. Phase shift is restricted to a 12 h window. Calculate the absolute difference of meta2d_phase between NRF and DRF and output phase shift when it is <= 12 h. Otherwise, phase shift is calculated by subtracting this absolute phase difference from 24, and output phase shift. If either of meta2d_pvalue is > 0.05, check which is < 0.05. If meta2d_pvalue of NRF is < 0.05, output meta2d_phase as the phase for NRF and denote DRF as arrhythmic (AR). If meta2d_pvalue of DRF is < 0.05, output meta2d_phase for DRF and denote NRF as AR. If both meta2d_pvalues are > 0.05, output AR for both tRF regimens.
4. Plot the 24 h profile of gene expression and phase shift in Prism (Figure 5).
  a. Two-way ANOVA is used for statistical analysis. Comparison between groups at individual time points is adjusted by Bonferroni correction. Statistical significance: ∗p < 0.05, ∗∗p < 0.01, ∗∗∗p < 0.001, ∗∗∗∗p < 0.0001. (Figures 5 and 6).

***Note:*** MetaCycle package is a recently developed N-version programming algorithm used in our study. Currently, there are many choices for both rhythmicity detection (e.g., JTK_CYCLE, eJTK, RAIN, CircaCompare, BIO_CYCLE, ZeitZeger, ECHO) and differential rhythmicity (e.g., LimoRhyde, DryR, CircaCompare). How to choose among these algorithms is an art, and can be referenced in excellent reviews (Wu et al., 2020; Hughes et al., 2017). In our study, we use MetaCycle for initial rhythmicity detection (Table S4), and validate alterations of rhythmicity parameters such as phase and amplitude by CircaCompare (Table S5, Figure 5).

***Note:*** When analyzing diurnal profiles entrained to light-dark cycles, both minper and maxper values are set to 24. Minper and maxper denote the minimum and maximum period, respectively. When analyzing diurnal profiles under constant light, minper and maxper values are relaxed to 20 and 28, respectively. In female mice, constant light increases the period length of voluntary wheel running behavioral rhythm by 1–2 h, instead of abrogating the circadian oscillation (Xin *et al.*, 2021).

1. Criteria for data exclusion: exclude samples with a quantification cycle (Cq, also known as threshold cycle, Ct) of > Mean ± 3 standard deviation (SD).
2. Collate and process the raw data.
  a. Sort the qPCR data in an Excel table from NRF ZT0-1 to DRF ZT24-4, including the Cq value, the starting quantity (SQ, relative quantity based on the standard curve) of the sample and the Cq value of the standard curve.
  b. Normalize the SQ of gene expression to the mean SQ of the reference gene u36B4, and save as the raw data (see Table S1).
  c. Arrange the raw data for illustration in Prism software and rhythmicity analysis in MetaCycle package, respectively (see Tables S2 and S3).
3. Analyze 24 h transcript profiles by MetaCycle package in R/RStudio.
  a. Save Table S3 in Excel format as a csv file named table3.csv.
  b. Code for profiles collected under light-dark cycles (12 h/12 h):
    i. library(MetaCycle)
    ii. meta2d(infile="table3.csv", filestyle="csv",
      outdir="metaout", minper=24, maxper=24,
      timepoints=rep(seq(0, 24, by=4), each=4),
      cycMethod=c("JTK","LS"), outIntegration="onlyIntegration")
  c. Code for profiles collected under constant light:
    i. library(MetaCycle)
    ii. meta2d(infile="table3.csv", filestyle="csv",
      outdir="metaout", minper=20, maxper=28,
      timepoints=rep(seq(0, 24, by=4), each=4),
      cycMethod=c("JTK","LS"),
      outIntegration="onlyIntegration")
  d. Obtain parameters describing circadian rhythmicity of gene expression, such as p-value, phase, amplitude (AMP), and relative amplitude (rAMP), according to the results of MetaCycle package meta2d function (Table S4).
  e. Calculate phase shift according to p-value and the results of CircaCompare (Parsons et al., 2020) (Figure 7, Table S5).

## Limitations

It has been noted that hours of inverted feeding condition the response of circadian clocks in extra-hepatic tissues to inverted feeding (Stokkan et al., 2001). 4-h inverted feeding alters behavioral rhythms whereas 12-h inverted feeding does not alter behavioral rhythms (Acosta-Rodríguez et al., 2017; Xin et al., 2021). Zhang et al. reported that 4-h NRF regimen in mice severely impairs body temperature homeostasis at 21°C and below (Zhang et al., 2020). Simply reversing the biphasic dietary intake pattern by 12 h does not mimic the effects of inverted feeding as suggested by a recent study (Xie et al., 2020). We use the 12-h inverted feeding regimen in this study.

Light cycles might affect the entrainment of peripheral clocks by inverted feeding. Early on, it has been shown that inverted feeding entrains the liver-clock under both light-dark cycles and constant darkness (Schibler and Sassone-Corsi, 2002; Vollmers et al., 2009). However, inverted feeding comes with an inherent limitation in elucidating clock synchronization in free-running conditions, such as constant darkness. Inverted feeding imposes a conflict between circadian rhythms in peripheral tissues, particularly liver and adipose tissue, and those in the suprachiasmatic nucleus (SCN) master clock. Light intensity and cycles would modulate the response to inverted feeding if light and food apply interactive actions to peripheral clocks. Indeed, we found that constant light (LL) during inverted feeding facilitates food entrainment of circadian clocks in heart and kidney and restores circadian clock oscillation in adipose tissue (Xin et al., 2021). This is associated with an extended period in behavioral rhythms (Xin et al., 2021). Hamaguchi et al. showed that NRF restored the circadian rhythmicity of tissue clocks in male mice under LL for 5 weeks, the peripheral clocks of which are otherwise desynchronized (Hamaguchi et al., 2015). This effect is uncoupled from the spontaneous rhythm-disrupting effect of LL (Hamaguchi et al., 2015).

So far, mounting evidence has supported the notion that prolonged LL is detrimental to circadian coherence among peripheral clocks and the SCN master clock, and contributes to derangement of metabolic homeostasis (Rumanova et al., 2020). In light of the role of tRF in fixing the LL-induced impairment of circadian rhythm and metabolism, LL might be an alternative regimen to mute SCN-based time signaling and study food entrainment of tissue clocks without the influence from the SCN master clock. In addition, 2 weeks in the 12:12 h light-dark cycle is commonly practiced for animal quarantine and acclimation in animal facility. In this protocol, acclimation for one week in the 12:12 h light-dark cycle is sufficient to reproduce the results. To avoid disruption of circadian cycles during tissue collection, we used red lamps for illumination in the dark phase, and euthanize the mice before relocating to a lighting hood for dissection.

Light intensity might affect the entrainment of peripheral clocks by inverted feeding. We and others use 200 lux of LED light intensity in time-restricted feeding studies (Xin et al., 2021; Zhang et al., 2020). When we unintentionally analyzed food entrainment in male mice under 500 lux of LED light intensity, DRF male mice showed dampened rhythmicity of circadian clock transcripts in heart and kidney (Xin et al., 2021). This observation is not conclusive for the lack of a head-to-head comparison. Very recently, Manella et al. have reported similar findings that Arntl and Nr1d1 transcripts show decreased amplitude or rhythmicity in quadriceps, kidney, and heart in their control mice (male C57BL/6 Alb-Cre+) after a 30-day DRF, though light intensity is not shown (Manella et al., 2021). We recommend reporting light intensity for inverted feeding studies.

The sampling regimen at a 4-h interval (i.e., sacrifice four mice every four hours for 24 h) is commonly used in molecular biology of circadian rhythms, but this practice comes at the expense of less accuracy in phase estimation and sensitivity to outliers. Permitting budget and human powers, it is recommended to cover two days with a 2-h sampling frequency (Hughes et al., 2017).

In this study, inverted feeding (DRF) is compared to the control feeding regimen (NRF). Although NRF is a completely reverse regimen against DRF, eating without time restriction (ad libitum, AL) is commonly used in mouse studies. A complete experimental design would ideally be three groups, i.e., DRF, AL, and NRF. Several studies compared DRF and AL in mouse liver and other peripheral tissues (Vollmers et al., 2009; Manella et al., 2021). Regarding liver, all three studies including ours demonstrate that liver clock and hepatic diurnal transcriptome entrains to inverted feeding. Manella et al. examined clocks in liver, adipose tissue, lung, quadriceps, kidney, and heart and profiled transcriptomes in liver, adipose tissue, and lung in male mice. The findings are consistent with ours, that is, peripheral tissues exhibit distinct patterns of phase entrainment of diurnal transcriptomes to inverted feeding (Xin et al., 2021; Manella et al., 2021). Thus, a comparison of AL vs DRF provides strength to our study.

## Troubleshooting

### Problem 1

There is not enough space and resource for building light-tight cabinets. (before you begin, step 1)

### Potential solution

An alternative approach would be to assign an entire room to the experiment where lights can be adjusted while the mice are in their home cages. Sometimes, simply avoid night-time usage of the room unless red lamps are used for illumination.

### Problem 2

Light intensity is not adjusted to 200 lux during acclimation. (before you begin, step 1)

### Potential solution

Adjust the light intensity to 200 lux. Acclimate mice for 7 days under 12:12 h light-dark cycles before subjecting the mice to tRF regimens.

### Problem 3

In a pilot study, we did not randomize the mice when assigning to DRF/NRF groups. Body weight record indicated significant difference in the starting body weight between groups. Since body weight is an end point in our 36-day inverted feeding study, the unequal starting body weight is a concern in this case. (before you begin, step 2)

### Potential solution

Randomize mice into groups until the average body weight between groups is not different (as described above).

### Problem 4

During overnight animal dissection and tissue collection, one time point is missing due to mute of the alarm clock. (tissue dissection, step 3)

### Potential solution

Since we showed that the 36-day inverted feeding and 7-day inverted feeding have similar effects on phase entrainment of circadian clocks in liver, adipose, kidney, and heart (Xin et al., 2021). At least for studying these tissues from female mice entrained to 12:12 h light-dark cycles, collecting samples at the missing time point the next day could make up the experiment.

### Problem 5

In MetaCycle analysis, a common error in the beginning stage is as follows. (quantification and statistical analysis, step 3)

> Error in meta2d(infile = "table3.csv ", filestyle = "csv", :

> Please check 'infile' or 'timepoints', the column number of 'infile' should be one more than the length of time points. See the example data in this package.

### Potential solution

In most cases, it is due to a mismatch between the actual samples arranged in columns in the csv-format input table (i.e., infile) and the settings of “timepoints” in the code. For example, if the NRF ZT04-2 liver sample were excluded from the analysis, the NRF group should have excluded the column “04_2”, been saved in a separate csv file, and analyzed by a separate MetaCycle code. The timepoints parameter should be adjusted to match the actual columns as below.

> timepoints=rep(seq(0, 24, by=4), c(4,3,4,4,4,4,4)

## Resource availability

### Lead contact

Further information and requests for resources and reagents should be directed to and will be fulfilled by the lead contact, Min-Dian Li (mindianli@tmmu.edu.cn).

### Materials availability

This study did not generate new unique reagents.

### Data and code availability

The published article includes all datasets generated or analyzed during this study. Original/source data and code for Figures in the paper are available i.e., Mendeley Data https://doi.org/10.17632/mb25x9t4m7.1.

## Supplemental information

Table S1. Raw data to demo RT-qPCR results, related to step 2b in the quantification and statistical analysis section

Table S2. Input data for illustration in prism software, related to step 2c in the quantification and statistical analysis section

Table S3. Input data for MetaCycle analysis, related to step 2c in the quantification and statistical analysis section

Table S4. Results of MetaCycle rhythmicity analysis, related to step 3d in the quantification and statistical analysis section

Table S5. Results of CircaCompare rhythmicity analysis, related to step 3e in the quantification and statistical analysis section
